# A prospective study of the relationship between prediagnostic Human Papillomavirus seropositivity and HPV DNA in subsequent cervical carcinomas

**DOI:** 10.1038/sj.bjc.6600454

**Published:** 2002-07-02

**Authors:** E Sigstad, A K Lie, T Luostarinen, J Dillner, E Jellum, M Lehtinen, S Thoresen, V Abeler

**Affiliations:** Department of Pathology, The Norwegian Radium Hospital, Oslo, Norway; The Finnish Cancer Registry, Helsinki, Finland; Department of Medical Microbiology, Lund University, University Hospital at Malmö, Malmö, Sweden; The Janus Committee, The Norwegian Cancer Society, Oslo, Norway; Department of Infectious Disease Epidemiology, National Public Health Institute, Helsinki, Finland; The Cancer Registry of Norway, Oslo, Norway

**Keywords:** cohort studies, viral persistence, tumour virology

## Abstract

Several prospective studies with invasive carcinoma as endpoint have supported Human Papillomavirus as a cause of cervical carcinoma. However, the largest study used seroepidemiology and did not analyse presence of Human Papillomavirus DNA in the subsequent tumour. Linkage of serum bank registries and cancer registries had identified 196 women with a registered cervical carcinoma after donation of a serum sample. For the present study, biopsies for 127 cases could be located, verified to contain invasive carcinoma and be amplified by PCR. Three control women who had remained alive and without cervical carcinoma during an equal length of follow-up had been matched to each of the case women and tested for HPV antibodies. Presence of Human Papillomavirus DNA in the tumours was analysed by general primer and type specific PCR. HPV16-seropositive women had a relative risk of 4.4 (95% CI: 2.2–8.8) to develop cervical carcinoma carrying HPV16 DNA. By contrast, there was no excess risk for Human Papillomavirus 16-seropositive women to develop cervical carcinoma devoid of HPV16 DNA. Prediagnostic HPV16 seropositivity was strongly correlated with later HPV16 DNA positivity of the tumour (*P*<0.001) and prediagnostic HPV18 seropositivity correlated with HPV18 DNA in the tumour (*P*<0.03). The link between prediagnostic seropositivity and type of viral DNA in the cancer implies that the carcinogenic effect of infection with these viruses is dependent on persistent presence of type-specific viral DNA.

*British Journal of Cancer* (2002) **37**, 175–180. doi:10.1038/sj.bjc.6600454 www.bjcancer.com

© 2002 Cancer Research UK

## 

The most important risk factor for development of cervical carcinoma is infection with the oncogenic types of human papillomavirus (HPV). A series of cross-sectional case-control studies, based both on HPV DNA testing of tumours (Walboomers *et al*, 1999), measurement of serum HPV antibodies (Dillner *et al*, 1995), or both (Nonnenmacher *et al*, 1995; de Sanjose *et al*, 1996) have found a consistent and strong association between HPV and cervical carcinoma.

That the association between HPV and cervical carcinoma is causal is supported also by prospective studies, both with cervical intraepithelial neoplasia (Koutsky *et al*, 1992; Chua *et al*, 1996) and invasive cervical carcinoma as the end-point (Lehtinen *et al*, 1996; Shah *et al*, 1997; Vonka *et al*, 1999; Wallin *et al*, 1999). However, most prospective studies are based on limited numbers of cases. The largest study to date is a seroepidemiological study that contained 182 cases (Dillner *et al*, 1997). However, prediagnostic seropositivity and type of HPV DNA in subsequently occurring invasive carcinoma has not been previously performed. Combining seroepidemiological studies with HPV DNA analyses in prospective studies could be important for several reasons. For example, there are indications that prior or ongoing HPV infections may interact in a complex fashion in cervical carcinogenesis (Luostarinen *et al*, 1999; Silins *et al*, 1999). By comparing prediagnostic assessment of virus exposure with presence of virus DNA in the tumour, it could be possible to infer whether type-specific persistence of HPV is the predominant mode of action of HPV in cervical carcinogenesis or whether previous infections with different HPV types also could have an effect on the cervical carcinoma risk. Furthermore, although a series of validation studies have indicated HPV type-specificity of HPV serology, within-study validation of the test performance is optimal. In order to provide a more informative and reliable prospective study on the effect of HPV infection on the subsequent risk of cervical carcinoma, we expanded a previous longitudinal seroepidemiological study with HPV DNA analyses of subsequent carcinomas. For improved accuracy, the histopathological diagnoses were also re-evaluated.

## SUBJECTS AND METHODS

### Subjects

The study base consists of about 550 000 healthy women who donated blood samples to three population-based serum banks in Norway, Finland and Sweden (Table 1

**Table 1:**
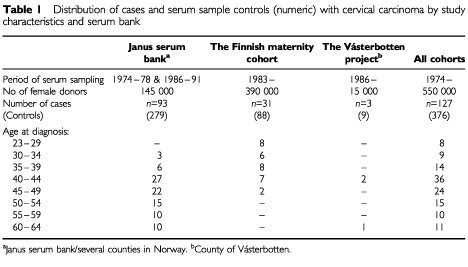
Distribution of cases and serum sample controls (numeric) with cervical carcinoma by study characteristics and serum bank

). The Janus project in Norway was initiated in 1973 to search in the premorbidity sera for changes indicative of chronic disease development at early stages and contains 425 000 blood samples from 294 000 subjects (stored at −25°C). This project was conducted in two phases. Phase I was conducted during the period from 1974 through 1978 and enrolled 29 000 subjects residing in one of three Norwegian counties (Finnmark, Sogn og Fjordane, or Oppland). Phase II was conducted during the period from 1986 through 1991 throughout Norway and enrolled 116 000 subjects. Whereas phase I enrolled subjects of a wide age range, phase II primarily focused on 40- to 42-year-old subjects. The response rate to the population-based invitation to participate was 85% in phase I and 75% in phase II (Jellum *et al*, 1995).

The Finnish Maternity Cohort contains blood samples from about 98% of all pregnant women in Finland. The samples are taken at the Finnish maternity clinics from women during early pregnancy (first trimester) for screening for congenital infections. Enrolment started in 1983 and is still ongoing. In 1993, the bank contained 710 000 samples obtained from 390 000 women (stored at −25°C) (Dillner *et al*, 1997).

The Västerbotten project (Sweden) was initiated in a county in northern Sweden in 1986 and is still ongoing. In 1993, it contained blood samples obtained from about 15 000 female residents (stored at −80°C). Each year all residents of ages 30, 40, 50, and 60 years are invited by letter to participate in a health-promoting project, including the donation of biologic samples for medical research. The response rate is usually about 65% (Dillner *et al*, 1997).

Women who had donated serum at least 2 weeks prior to a cervical malignant tumour diagnosis were identified by linkage of the data files of the serum banks with the nation-wide cancer registries in Norway, Finland and Sweden, using the personal identification numbers. Analysis of the HPV serology in the present study by lag time in tertiles (<12, 12–59 and >59 months between sampling and diagnosis) found no statistically significant evidence on dependence of lag time (Dillner *et al*, 1997). Notification of new cancer cases is compulsory in the three countries, and multiple data sources ensure almost 100% completion of the cancer registries (Engeland *et al*, 1993). From start-up of the respective serum banks until the linkages done in 1994, invasive cervical carcinoma had been diagnosed in 196 women with a serum specimen stored. In addition, 24 women who developed cervical malignant tumour had been sampled, but had no longer had serum stored.

Of these, 10 cases were excluded: four who had a lag time between enrolment and diagnosis of less than 2 weeks, two for whom the serum samples were not located, two cases for whom the available biopsies had been taken 2 years after the first diagnosis of invasive carcinoma was made and two cases where the blocks were not correctly located. From the rest we were able to retrieve histological material from 153. Twenty-six of these were excluded: one with an unclassifiable malignant tumour, one small cell carcinoma, two cases with benign histology from another organ, 10 cases with tumour of noninvasive histology (carcinoma *in situ*), six cases that could not be amplified by PCR in the HLA test, five cases with an empty control slide and one case where the PCR detection for HPV was done on incorrect material. The registry linkage is the same as in the previous study (Dillner *et al*, 1997), but more cases are excluded due to the fact that retrieval of tumour specimens, amplifiability in PCR and verified histopathological diagnosis on review was also required in this study.

The distribution of the different types of cervical carcinoma and age at diagnosis of the remaining 127 cases are shown in Table 2

**Table 2:**
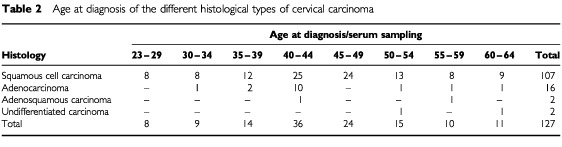
Age at diagnosis of the different histological types of cervical carcinoma

. More than two thirds of the patients developed cervical carcinoma before the age of 50 years and more than 10% were younger than 35 years. If more than one prediagnostic serum sample was available, the first (oldest) sample was chosen. For each case patient, three control subjects who had remained alive and free of cervical malignant tumour for an equal length of follow-up were selected and individually matched for sex, age at serum sampling (±2 years), length of serum storage (±2 months), country, and in Norway also for county of residence. If three control subjects per case patient fulfilling the matching criteria could not be found, the matching criteria on age at serum sampling and length of serum storage were widened in successive steps of 1 year of age and 1 month of storage until three control subjects were found. The mean difference in age was 1.5 years. The maximum age discrepancy (4 years) was present for three of 376 control subjects. The mean difference in time of sampling was 2.1 months, with none of the control subjects differing by more than 6 months. The prospective study was approved by the Data Protection/Institutional review boards of the respective countries.

### Laboratory methods

All laboratory analyses were performed on coded specimens.

### HPV serology

Capsids containing both the L1 and L2 capsid proteins of HPV type 16 (clone 114/K), 18, or 33 or of bovine papillomavirus (BPV) type 1 generated in insect cells by use of recombinant baculoviruses were the kind gift of Dr John T Schiller, National Cancer Institute, USA and Dr Martin Sapp, University of Mainz, Germany. The detection of human serum antibodies was done in the standard enzyme-linked immunosorbent assay method employing a monoclonal antibody against human immunoglobulin G (IgG) (γ chain) and a goat anti-mouse IgG horseradish peroxidase conjugate (Dillner *et al*, 1995). For each serum, we calculated the difference in optical density obtained with plates coated with intact HPV capsids and plates coated with control antigen (disrupted BPV capsids). The cutoff levels for determining seropositivity from continuous values were established in previous work (Andersson-Ellström *et al*, 1996), which had determined that a cutoff level of 0.100 absorbance unit was able to distinguish HPV-infected women from virgin women. Our previous report had used two alternative cut-off level designations and had found little differences in results (Dillner *et al*, 1996) and in this study only the 0.100 cutoff was used. All assays were performed at least twice. The proportions of samples with discrepant results upon repeated analysis were 1.9% for HPV 16, 7.2% for HPV 18, and 4.3% for HPV 33. Discrepancies were resolved by multiple repeat analyses. A panel of serum samples from virginal women was analysed in parallel and no HPV antibodies could be found (Andersson-Ellström *et al*, 1996).

### Histologic review

All histologic specimens were reviewed by two of the authors, E Sigstad and V Abeler, who were blinded to registered diagnoses and to all other results in the study database. The tumours were classified and graded according to the World Health Organisation (WHO) histologic typing of female genital tract tumours (WHO, 1975). When possible, the tumour was classified on the first biopsy. When the first biopsy was of bad quality or the material was too small, the classification was done on the best-preserved specimen. All slides from each case were stained with standard haematoxylin and eosin staining. In some cases preoperative irradiation had affected the cervical tumour to the extent that classification was difficult.

### HPV DNA detection

DNA was extracted from paraffin-embedded tissue as described by Shibata *et al* (1988) but instead of boiling, the tissue sections were treated with proteinase K and incubated at 55°C overnight. Proteinase K was then inactivated at 95°C for 15 min. The microtome was cleaned in xylene and a new blade was used between each block. Sections from empty blocks were used as a contamination control in-between each specimen.

HPV–PCR was performed with the L1 consensus primers Gp5+/6+, giving a PCR product of 150 bp (de Roda Husman *et al*, 1995). Amplification was carried out for 40 cycles in a Perkin Elmer Cetus thermal cycler. Each cycle of amplification consisted of 1 min of denaturation at 94°C, 1 min of annealing at 43°C, and 1 min of polymerisation at 72°C. The first cycle was preceded by 7½ min denaturation and the last cycle was extended by a 7 min elongation step at 72°C. The HeLa cell line was used as a positive control and H_2_O as a negative control in each PCR assay. The quality of the DNA extracts was tested by amplification of HLA-DQ1 with the primers GH26/GH27, giving a PCR product of 250 bp. PCR products were controlled by gel electrophoresis (10% polyacrylamide), followed by transillumination with UV-light. Specimens not giving a DQA1 product, but giving a Gp5+/6+ product, went to further examination, while specimens not giving products were excluded from the HPV–PCR.

HPV DNA positive cases were typed with E6 and E7 type-specific primers for HPV16, 18 and 33 (Hagmar *et al*, 1992; Lie *et al*, 1999). All HPV DNA negative cases were also tested with the HPV16 and 18 primers to control for possible false negatives due to deletion in the L1 region after integration of HPV.

### Statistical analyses

The odds ratios (ORs) and their 95% confidence intervals (CI) were estimated by conditional logistic regression (Breslow and Day, 1980) with the EGRET software (Cytel Software Corporation, Cambridge, MA, USA). If the asymptotic model did not converge, analyses were conducted with the exact inference methods (Mehta *et al*, 1985) for contingency tables implemented in the EPIXACT software (Cytel Software Corporation, Cambridge, MA, USA). Fisher`s exact test tested for equity between proportions. A two-tailed P value of less than 0.05 indicated statistical significance.

## RESULTS

HPV16 seropositive women had an increased risk to develop invasive cervical carcinoma compared to HPV16 seronegative women (OR=2.4; 95% CI 1.4–4.3), which was a finding similar to our previous report from the same material (Dillner *et al*, 1997). The risk to develop squamous cell carcinoma was somewhat higher (odds ratio=2.8; 95% CI 1.6–5.0) (Table 3

**Table 3:**
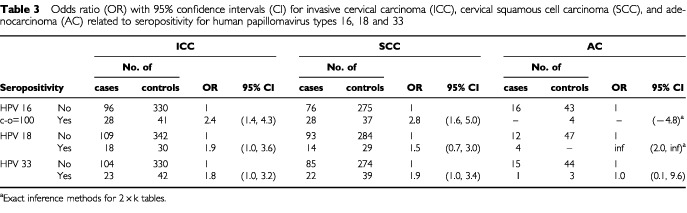
Odds ratio (OR) with 95% confidence intervals (CI) for invasive cervical carcinoma (ICC), cervical squamous cell carcinoma (SCC), and adenocarcinoma (AC) related to seropositivity for human papillomavirus types 16, 18 and 33

). HPV18 and HPV33 seropositive women both had two-fold relative risks to develop cervical carcinoma. Because of small numbers, the relative risk of HPV18 seropositive women to develop cervical adenocarcinoma could not be estimated, but the risk was highly significantly increased (Table 3). Joint seropositivity for both HPV16 and 18 further increased the risk for invasive cervical carcinoma (OR=3.5; 95% CI: 1.6–7.6).

HPV16-seropositive women were at a 4.4-fold risk to develop HPV16 DNA carrying invasive cervical cancer, but they had no excess risk to develop cervical carcinoma containing other HPV types or carcinomas negative for HPV DNA by PCR (Table 4

**Table 4:**
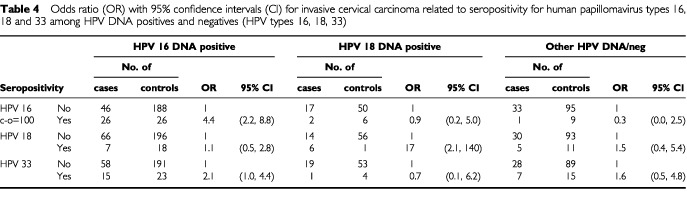
Odds ratio (OR) with 95% confidence intervals (CI) for invasive cervical carcinoma related to seropositivity for human papillomavirus types 16, 18 and 33 among HPV DNA positives and negatives (HPV types 16, 18, 33)

). Restricting the analysis to cervical squamous cell carcinoma gave similar results (Table 5

**Table 5:**
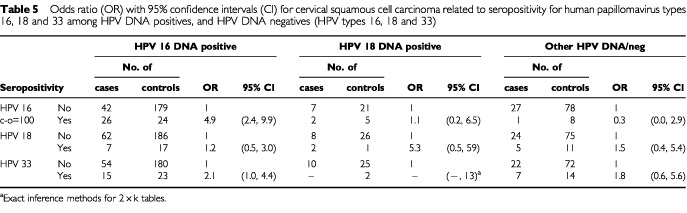
Odds ratio (OR) with 95% confidence intervals (CI) for cervical squamous cell carcinoma related to seropositivity for human papillomavirus types 16, 18 and 33 among HPV DNA positives, and HPV DNA negatives (HPV types 16, 18 and 33)

).

Among 29 HPV16 seropositive women who developed invasive cervical carcinoma, 26 had HPV16 DNA positive tumours whereas 46 of 96 HPV16 seronegative women developing carcinoma had HPV16 DNA positive tumours (*P*<0.001). For squamous cell carcinoma the corresponding figures for HPV16 DNA positivity were 26 of 29 HPV16 seropositive, and 42 of 76 HPV16 seronegative women, respectively (*P*<0.001).

HPV18 DNA positivity was found in six of 18 HPV18 seropositive women with invasive cervical carcinoma and in 14 of 110 HPV18 seronegative women (*P*=0.027).

Only one woman had a cervical carcinoma biopsy containing HPV33. She was HPV33 seronegative in her prediagnostic serum sample. In contrast to the results for HPV16 and HPV18, HPV33 seropositivity was associated with increased risk to develop carcinoma containing other viral types: A two-fold relative risk to develop HPV16 DNA positive cancers was seen (Table 4).

## DISCUSSION

This prospective and population-based study enabled an investigation of the natural history of cervical carcinoma with regards to HPV exposure and to intermittent and persistent infections with HPV. The present study advances previous studies on prediagnostic seropositivity in that it also correlates seropositivity to HPV DNA positivity in specimens from paraffin blocks histologically shown to contain a subsequently developed invasive cervical carcinoma. Histopathological specimens were provided from 153 cases out of 196 women reported with a diagnosis of cervical carcinoma after enrollment. Re-examination of these cases by two of the authors confirmed the diagnosis of cervical carcinoma in most cases. However, as many as 26 cases (17%) were excluded. Twelve of these (7.8%) were excluded because the diagnosis of invasive cervical carcinoma was not confirmed. This is in line with what is shown by others (Chua *et al*, 1996) and demonstrates the importance of histological re-examination in registry-based studies.

There were significantly more HPV DNA positive tumours among the seropositive compared to the seronegative cases. The results confirm earlier studies demonstrating the correlation between infection with the investigated types of HPV16 and HPV18 and an increased risk of developing cervical carcinoma harbouring HPV16 or HPV18.

Prediagnostic seropositivity for HPV raised the risk to develop cervical carcinoma only for those carcinomas that contained the same type of viral DNA. An exception to this was HPV33 seropositivity that was associated with an increased risk to develop HPV16 DNA-carrying carcinomas. There are several possible reasons for this finding, notably (i) chance, (ii) cross-induction of HPV33-specific antibodies by infection with the closely related HPV16 and (iii) a biological interaction whereby previous HPV33 infection promotes the oncogenicity of HPV 16. The fact that a similar tendency for HPV33 to increase the cervical carcinoma risk together with HPV16 has been noted before (Silins *et al*, 1999) suggests that the phenomenon is not merely due to chance.

HPV serology has high specificity for sexually transmitted HPV types, since HPV seropositivity is rare among virginal or monogamous women (af Geijerstam *et al*, 1999). However, a series of validation studies have concluded that only about 50–70% of genitally infected women (as determined by PCR) will seroconvert (Kirnbauer *et al*, 1994; Carter *et al*, 1996; Kjellberg *et al*, 1999). The reason why HPV serology is not very sensitive for detection of HPV infection is not known. In the present study only 26 out of 72 (36%) of women with HPV16 DNA-positive carcinomas had prediagnostic HPV16 seropositivity and only 5 out of 19 (26%) of women with HPV18 DNA-positive carcinomas had prediagnostic HPV18 seropositivity. There are several possible reasons why the sensitivity was particularly low in our study. There was a long time-lag between drawing of the serum sample and the occurrence of the carcinoma and some women may have acquired their infection after the drawing of the serum sample. The serum samples had been stored at −20°C for up to 20 years and although IgG is generally stable on storage, the possibility that there may have been some decay on prolonged storage can not be excluded.

There were a few cases with no HPV DNA in tumour. This could represent false negative HPV DNA tests due to old tissue samples or samples of bad quality. Since most of the HPV infections are transient (Hildesheim *et al*, 1994; Evander *et al*, 1995; Ho *et al*, 1998), another possibility is that after the infections with HPV 16, 18 and/or 33 have ceased, a history of HPV infection may still increase the risk for cervical carcinoma. However, as seropositive women were not at increased risk to develop carcinomas that were HPV-negative or containing other types of virus, our data strongly suggests that persistent type-specific HPV infection is required and that the HPV DNA-negative cases either really are HPV-negative or contain unknown HPV types that are not adequately amplified by the PCR tests used.

In conclusion, this study advances previous prospective epidemiological studies of HPV and cervical carcinoma by providing improved case definition with reclassified histological diagnoses and by providing a within-study evaluation of the sensitivity of HPV serology, enabling correction of the relative risk estimates for misclassification. Furthermore, the finding that prediagnostic seropositivity only increases risk for development of carcinomas with the same type of HPV DNA in the tumour further supports the view that the role of HPV infection in cervical carcinoma is mediated via persistent type-specific HPV infection.
